# Keyword Index for Volume 103

**DOI:** 10.1038/sj.bjc.6606039

**Published:** 2010-12-07

^1^H MRS 1297

4-hydroxynonenal 1263

5-fluorouracil (5-FU) 340, 581, 1163, 1617 5-year lung cancer risk 423

A1 domain of tenascin-C 827 ABCB1 560 ABCG2 567 acne 411 acrylamide 1749 active drug combinations 1369 adenocarcinoma 217 adenovirus 1822 adjusted calcium 870 adjuvant chemoradiotherapy 1163 adjuvant chemotherapy 1788 adolescent cancer 1448 advanced solid tumours 993, 1554 age 112, 416, 970 aggressive fibromatosis 482 ago2 532 albumin 870 ALCAM 1048 alcohol consumption 127, 741, 747 ALKBH2 362 allogeneic bone marrow transplantation 1597 aminopeptidase inhibitor 1362 AML 275, 1292 AMPK 1025 anal carcinoma 1858 anastomotic leakage 970 androgen receptor 1001 angiogenesis 1, 1422, 1571 angiopoietin-2 1407 angiotensin I-converting enzyme inhibitors 1644 angiotensin II type-1 receptor blockers 1644 Angiotensin-2 1698 ANO1 715 anthracycline 1518 anti-androgens 1001 antiangiogenesis 18, 52, 462, 772 antibody–drug conjugate 676 anticancer drugs 1815 antineoplastic drug 239 anxiety 1502 Apo2L 1783 apolipoproteins 391 apoptosis 36, 43, 239, 256, 354, 642, 1237, 1692, 1783, 1808 arginine 954 arginine deiminase 954 argininosuccinate synthetase 954 aromatase inhibitor 291 arsenic 1277 arthralgia 291 ASK1 1380 association study 575 AT1-R 1698 AT2-R 1698 ATP 656 autoimmune conditions 112 autophagy 1209, 1580 avoidable deaths 1109 axitinib 1554

*β*_1_-integrin 1710 base excision repair 1875 basic fibroblast growth factor 370 BCAR4 1284 Bcl-2 668, 1710, 1808 Beclin 1 1209 BelCaP 12 belinostat 12 betulinic acid 43 bevacizumab 772, 1407, 1524, 1529, 1536 Bik 1808 biliary stones 115 biliary tract cancers 115, 469 biomarkers 232, 701, 708, 1313, 1407, 1794, 1858 bioreductive 201 BMI 741, 1443, 1467 bone microenvironment 370 bone tumour 73 BP-1 peptide 82 brain cancer 1103, 1606 *BRCA2* 918 BRCA2 mutation 1192 breaking bad news 171 breast cancer 82, 90, 249, 291, 297, 401, 475, 524, 560, 668, 759, 772, 899, 1034, 1040, 1076, 1097, 1103, 1201, 1400, 1443, 1749, 1788, 1794, 1831, 1835 breath 542 Burkitt's lymphoma 265, 1736

C35 401 CA 19-9 tumour marker 1318 CA-125 1657 CaCC 715 calcifications 1034 cancer inequalities 1076 cancer network 1076 cancer prevention 1453 cancer screening 708 cancer stem cell 382, 439, 1692 capecitabine 617, 845, 1524, 1680 carbonate 1034 carboplatin 12 carcinogens 90 carcinoma 1432 carcinoma ex pleomorphic adenoma 1846 carcinoma *in situ* 1269 carcinoma, renal cell 796 carcinoma, squamous cell 1462 carrier risk estimates 1875 case–control 411, 1729 CD133 382 CD147 1008 CD166 382 CD44 382 CD44v3-10 1008 CD70 676 CD95 642 CEBPA 275 cell cycle 18, 239, 354 cell growth 256 cell lines 340 cell motility 370, 715 cell signalling 223 cell-cycle arrest 1237 cellular senescence 505 cerebellum 1297 cervical cancer 209, 310, 613, 939, 1773 cervical screening 209, 939 cetuximab 1542 CGH 1277 chemoradiotherapy 1349 chemorefractory 324 chemoresistance 73, 656, 1822 chemosensitivity 332, 656 chemotherapy 6, 159, 1407, 1415, 1644 childhood cancer 136, 149, 1122, 1448, 1663 childhood leukaemia 1122, 1128 China 1085 CHR-2797 1362 chromatin 1269 chronic disease 1742 chronic lymphocytic leukaemia 642 cisplatin 469, 845, 1343 clinical skin examination 1502 clinical trials 285, 1313 clonal evolution 439 CNS 1588 cohort study 1093, 1443, 1749, 1755 colon cancer 730, 961, 1025, 1209, 1415, 1680, 1852 colorectal cancer 120, 159, 315, 324, 340, 382, 505, 575, 581, 747, 787, 910, 970, 975, 1356, 1407, 1453, 1496, 1536, 1627, 1755, 1765, 1875 colorectal liver metastases 1542 combination chemotherapy 469, 796, 993 combined modality treatment 1524 communication skills 171 comorbidity 1742 complement system 1201 computerised assessment 1489 copy-number variation 1277 cost-effectiveness analysis 1773 COX-2 975, 1263 CP-868,596 1554 C-reactive protein 870, 1154 cross-talk 1001 CRP 101, 1649 CTL 552 curative surgery 970 CXCL12 1671 CXCR4 1671 cyclooxygenase 1182 CYP2D6 765 CYT997 597 cytokines 291, 1453 cytology 310, 939 cytotoxicity 239, 1369

DCE-MRI 597 DCIS 1034 death receptors 1380 dendritic cells 1415, 1597 deprivation 446 diabetes 115, 120 diagnostic marker 217 dicer 532 diet 1749, 1760 dietary fatty acid 1182 differentiated thyroid carcinoma 1115 disabled-2 1716 discriminative markers 1048 discriminatory power 423 disease-specific survival 899, 1831 dissemination 1706 distant metastasis 715 distress screening 1489 DNA cytometry 1432 DNA double-strand breaks 1822 DNA repair 36, 362, 1822 DNA replication licensing 701 DNP 1400 docetaxel 332, 560, 1201, 1343, 1554 donor lymphocyte infusion 1597 dose finding 1529, 1815 dose-dense 6 doxorubicin 178 drug resistance 607, 1139, 1369, 1671 ductal carcinoma *in situ* 94 DUSP 265 dynamic contrast-enhanced magnetic resonance imaging 486 dyslipidaemia 617

*E. coli* 975 early breast cancer 776 early diagnosis 223 E-cadherin 249, 1835 economic evaluation 315 educational position 1109 EGFR 510, 622, 1019, 1765 EGFR inhibitor 347 elastin-derived peptides 1562 EMT 1716 endocrine therapy 1284 endometrial cancer 812, 889, 933 endometrial cancer risk 127 energy balance 1025 enzastaurin 802 EpCAM 382 epidemiology 136, 933, 947, 1724, 1729, 1760 epidermal growth factor 510 epigenetics 265, 1269 epirubicin 1201 epistaxis 772 epithelial dysplasia 1432 epithelial to mesenchymal transition 401 epothilones 1548 Epstein–Barr virus (EBV) 1736 ERBB2 1284 ERBB3 1284 ERBB4 1284 ERK 1025 ERK1/2 510 erlotinib 622 esophageal cancer 1085 ethnicity 143, 1448 *EVI1* 1292 Ewing's sarcoma family of tumours 370, 1380 excesion repair cross- complementation group 1 (ERCC1) 845 exercise 1760 experimental animal models 61 extracellular signal-regulated kinase 510

family history 1502 FAP 910 fenretinide 1380 FHIT 802 figitumumab (CP-751,871) 332 first-line treatment 1536 flavonols 1453 Flt-1 82 fludarabine 642 fluorescence *in situ* hybridisation 1335 foetal germ cells 1269 FOLFIRI 1536 folinic acid 1163 follow-up 1657 follow-up compliance 310 food diary 747 food frequency questionnaire 747 formalin-fixed tissue 663, 1229 French Polynesia 1115 FTIR 1034 fumarate 1400

GalCer 524 gastric cancer 1182, 1343 gastrointestinal stromal tumour (GIST) 165 GC-MS 542 gemcitabine 52, 469, 1529, 1644 gene expression 656, 1852 gene expression profiling 1788 gene methylation 820 genetic testing 918 genetics 685, 1502 genomic instability 1139 genomics 759 genotoxicity 1263 GGT 90 GIST 1422 glioblastoma 498, 827, 1606 glioma 29 GPS 1356 graft *vs* host disease 1597 graft *vs* tumour reaction 1597 granulocyte-macrophage colony-stimulating factor 1331 Great Britain 430

HAART 416 HDAC 12 HDAC inhibitor 1680 HDAC4 1215 head and neck cancer 1173 health policy 446 hedgehog 43 hepatocellular carcinoma 837, 954, 1215, 1617 HER2 401, 475, 552, 663, 889, 1331, 1788 HER2 and ER-negative breast cancer 1048 HER-2 level amplification 1335 heterogeneity 1139 hI-con1 812 HIF-1 1, 1571 HIF1*α* 1209 high-resolution melting analysis 1627 histone deacetylase inhibitor 1391 histopathology 94 HIV 265, 416 HNSCC 715, 1245 Hodgkin's lymphoma 1081 Hospital Episode Statistics 143 HPV DNA testing 939, 1773 HRQOL 1173 *hTERT* 1255 human breast cancer xenograft 1192 human papillomavirus 209, 310, 1510 Hybrid Capture 2 939 hypertriglyceridaemia 617 hypopharyngeal squamous cell carcinoma (HSCC) 877 hypoxia 201, 1057, 1571

Id proteins 1237 IF protein 82 IGF 1479 IGF-I 132, 1089 IL-17 1245 imaging 1657 imatinib mesylate 482 immunisation 209 immunocytokines 827 immunohistochemistry 899, 1831 immunotherapy 812, 1415 *in vivo* 1066, 1562 incidence 132, 143, 165, 416, 947, 1089, 1443, 1448, 1462 Indians 143 infants 1724 inflammation 1356 inflammatory breast cancer 532 infliximab 1149 insulin 1479 insulin-like growth factor 132, 1089 insulin-like growth factor type 1 receptor (IGF-IR) 332 insulin-like growth factor- binding protein 7 1606 intelectin 517 interferon 1163 interferon-*α* (IFN-*α*) 1617 interleukin-2 827 interleukin-6 787, 1154 interobserver reproducibility 1057 intrinsic subtype 249 invasion 567, 802, 1182, 1422 ionising radiation 186 irinotecan 581, 993, 1325, 1524, 1529 ITAM 401

JNJ-26483327 987

kallikrein 1422 kinase mutations 165 KIT 165 KRAS 1019, 1627, 1765

laryngeal cancer 186 LDH 1209 leucovorin 1343 leukaemia 149, 1663, 1724, 1729 leukaemia stem cell 439 lipids 617 liver cancer 741 liver function tests 870 liver resectability 1542 lobular carcinoma 1097 Luminex 1221 lung cancer 217, 354, 423, 727, 1093, 1103, 1144, 1277, 1325, 1692, 1870 lymph node density 613 lymphocyte 685 lymphoma 149, 1663 Lynch syndrome 1840

macrophages 629 magnetic fields 1122, 1128 malaria 1736 malignant mesothelioma 629 malnutrition 1736 MammaPrint 1788 MAPK 1716 Markov Chain Monte Carlo 430 Mcm5 701 Mcol-A 1562 MDM2 186 mediation modelling 115 medulloblastoma 1297, 1588 melanoma 820, 1229, 1502, 1548, 1562 menarche 1760 mesenchymal stem cell 1692 mesothelial cells 517 mesothelioma 430, 517, 885 meta-analysis 127, 1128, 1467, 1875 metabolites 1297 metachronous metastases 159 metastasis 196, 201, 324, 524, 802, 861, 1008, 1066, 1182 metastatic breast cancer 607, 765, 1331, 1518 metastatic colorectal cancer 1524, 1529 metastatic renal cell cancer 1154 methylation 1846 metronomic chemotherapy 52 MGMT 29, 820 MHC class I 552 microarray 685 microRNA 532, 877, 885, 1144 microRNA biogenesis 1870 microsatellite instability 1840 migrants 143 migration 567, 1040 MiR-1-2 1292 MiR-133a-1 1292 miR-21 1617 miR-22 1215 *miR-29a/b/c* family 275 *miR-489* 877 miR145 256 miRNA 567 miRNA profiles 693 mismatch repair deficiency 340 MMP 1066 mobile phones 1729 mode of presentation 970 molecular imaging 1606 molecular marker 524, 1858 molecular subtypes 1097 monitoring 693 monocarboxylate transporters 1008 monoclonal antibodies 332, 1765 mortality 430, 727 motility 82 mRNA 532 MSH6 1840 mTOR 622, 649, 1001 mTOR inhibitor 1637 mucoepidermoid carcinoma 510 multidrug resistance 178, 1008 Multiethnic Cohort 120 multiple myeloma 1580, 1808 *MUTYH* 1875 myeloid differentiation 629 myeloid-derived suppressor cells 629 nanoparticles 1606 nanosensor 542 nasal septum perforation 772 NBS1 1822 necrosis 1057 nedaplatin 1325 needle biopsy 1706 needle track 1706 neoadjuvant chemoradiotherapy 1019 neoadjuvant chemotherapy, chronotherapy 1542 neoadjuvant endocrine therapy 759 neo-adjuvant therapy 297, 1794 neoplasms 735, 1462 neuroblastoma 1597 neuropathy 1580 never smokers 1277 NGR-hTNF 837 NLCQ-1 (NSC 709257) 201 nomogram 1649 non-small cell lung cancer 36, 347, 1221, 1325 NOX4 1040 nuclear test 1115 nucleoside analogue 1391 Nutlin-3 186

*O*^6^-methylguanine-DNA methyltransferase 498 oesophageal cancer 232, 552, 845, 1349 oesophagus 735 oestrogen receptor 475, 759 oncogene 1144 oral cancer 303 oral contraceptives 1755 oral potentially malignant disorders 303, 432 oropharyngeal SCC or oropharyngeal cancer 1510 osteopontin 1048, 1229 osteosarcoma 73 ovarian cancer 61, 656, 676, 685, 693, 1103, 1237 ovarian cancer recurrence 1657 ovarian cancer screening 454 oxaliplatin 1349, 1415 oxidative stress 90, 1808

*P. acnes* 411 p38^MAPK^ 1380 p53 186 p70S6K 649 paclitaxel 12, 889, 1362 palliation 1318 pancreatic 676 pancreatic adenocarcinoma 391 pancreatic cancer 52, 223, 649, 1057, 1318, 1571, 1644, 1671 Pap smear 1773 PARP 1588 participation 1496 parvin-*β* 852 pathological response 297, 1335 patient reported outcome measure 1485 patient satisfaction questionnaire 1485 patients’ attitudes 1801 PDGFRA 165 pediatric cancer 1663, 1736 pegylated liposomal doxorubicin 1518 peptide aptamer 1237 personalised medicine 1144, 1852 PET 201 P-glycoprotein 178 pharmacodynamics 987 pharmacogenetics 765 pharmacokinetics 560, 597, 987, 993, 1548 phase I study 462, 597, 607, 987, 1362, 1637 phase Ib 1554 PHD2 1 PHD3 1571 phenotype 765 phosphatase and tensin homologue (PTEN) 1617 phospho-extracellular signal-regulated kinase 223 photodynamic therapy 362 Photofrin 362 physical activity 933 physical exam 1657 PI3K 370 PLA 663 pleural effusion 517 PlGF 82 polyethylene glycol 954 polymorphisms 560, 581, 1870 pooled analysis 1128 population 475 population-based 735, 1663 POSSUM 1356 power lines 1122 PP2A phosphatase 462 preclinical model 1192 precursor lesion 1085 predictive factors 29, 297, 482, 1562 predictive marker 1765 predictive multiscale model 486 predictive value of tests 708 premenopausal 90 pre-operative 1649 prognosis 94, 101, 613, 668, 918, 961, 1025, 1215, 1229, 1627, 1649, 1835, 1852, 1858 prognostic factors 159, 852, 1057, 1318, 1698 prognostic marker 249 prognostic model 776 programmed cell death 4 (PDCD4) 1617 progression 961 prolactin 1097 proliferation 1215 prospective study 730, 747 prostate cancer 256, 411, 462, 701, 708, 918, 1008, 1066, 1103 prostate-specific antigen 708 proteasome inhibitors 1580 protein–protein complexes 663 proteomics 101, 232, 391 PSA 701 psychosocial distress 1489 PTK6 (BRK) 663 *PTPN11* 877 puberty 1760 pyruvate 1400 quality of life 1318 quantitative real-time PCR 899 quercetin 642 quitting 1093

Rac1 370 RAD001 622 radiation 347, 1325 radiation-induced cancer 1115 radiation-related breast cancer 1081 radioembolisation 324 radiofrequency 1729 radiotherapy 6, 201, 1489, 1510 randomised controlled trial 310 randomised study 171 *Ras* mutations 505 RASSF1 802 RCC 101, 1649 RCTs 1801 reactive oxygen/nitrogen species 1263 receptor tyrosine kinase 196 receptor, epidermal growth factor 796 rechallenge 1518 rectal cancer 730, 1019, 1163 recurrence 776 recurrent neuroblastoma 1369 relapse risk 759 relative risks 423 relative survival 446 renal cell carcinoma 1149, 1255, 1698 renal insufficiency 1815 renin–angiotensin system 1644 replication error 340 reproductive history 1755 resistance 178 response to therapy 232, 1201 rhabdomyosarcoma 43 risk assessment 1788 risk factors 303, 411, 1085 risk model 423 risk predisposition 575 risk stratification 1144 RNA expression profile 73 RNA interference 354 RNAi 975 ROS 1040 RT–PCR 656

SAA 101 sagopilone 1548 salivary gland tumour 510, 1846 sapacitabine 1391 screening 303, 310, 1496, 1773 scrotum 1462 second breast cancer 1081 sedentary behaviour 933 seeding 1706 SELDI 101 selective internal radiation therapy 324 selenium deficiency 1736 self-renewal 439 sentinel node biopsy 1229 serum biomarkers 223, 391, 1221 side population cells 567, 1692 signal transduction inhibitors 1001 siltuximab 1154 single-domain antibody 1606 sirolimus 649, 796, 1637 SIRT 324 skin neoplasm 112 skin self-examination 1502 small cell lung cancer 622, 1710 smoking 120, 727, 1093 smoky coal 727 smoothened receptor 1297 SNP (single-nucleotide polymorphism) 575 socioeconomic status 136, 303, 446, 741, 1076, 1109, 1496, 1742 sodium selenate 462 soluble interleukin-6 receptor 787 sorafenib 1149, 1637 squamous cell carcinoma 217 squamous cell carcinoma of the head and neck 186 Src kinase 899, 1831 Sri Lanka 303 stroma 1040 sunitinib 196, 993 surgery 6, 285, 1510 survival 149, 475, 861, 889, 970, 1076, 1109, 1335, 1356, 1663, 1742, 1870 survival analysis 776, 1627 survival rate 1462 survivin 36 SV40 885 Switzerland 416 Syk kinase 401 synchronous metastases 159

tamoxifen 765 tamoxifen resistance 1284 targeted gene therapy 1822 targeted therapy 622 TEF 1808 tegafur/uracil 1343 telomerase activity and pathways 1255 temozolomide 29, 498, 820, 827, 1588 temsirolimus 649 testicular cancer 1269, 1467 testicular germ cell tumour 18 TGF*β* 1716 Th17 1245 therapeutic response 486 therapeutic target 1671 therapy 196, 239, 1479 therapy toxicity 820 thymic carcinoma 196 thymidine phosphorylase (TP) 845, 1680 thymidine synthase (TS) 354, 845, 1680 thymoma 6, 196 time-dependent variable 727 tissue microarray 961, 1710 TNFSF10 256 tobacco use 1093 tosedostat 1362 toxicity 581, 772, 1548 TP53 362 TRAIL 642, 1380, 1692, 1783 transcriptional regulation 275 transgenic mice 1297 trans-signalling 787 trastuzumab 61, 297, 1331, 1335 treatment outcome 505 treatment pathways 315 treatment response 1400 triage 939 triglycerides 617 triple-negative breast cancer 249 tumour cells 1706 tumour growth model 486 tumour heterogeneity 439, 486 tumour marker screening 693 tumour microenvironment 52, 1245 tumour suppressor 1144 tumour-infiltrating lymphocytes 1245 tylosin 178 tyrosine kinase inhibitor 18, 987, 1637

ubiquitin D 961 UGT8 524 UHRF1 217 UKCTOCS 454 unresectable 6 u-PAR 802 upper urinary tract 852 urinary cancers 852, 812, 1103 uveal melanoma 285

vaccination 1415 vaginal cytology 1657 valproate 1391 vascular endothelial growth factor receptor-2 (KDR) 18 vascular-disrupting agents 597 vascular-targeting agent 837 VEGF 1529 VEGFR inhibitor 347 venous thromboembolism 947 viral hepatitis 741 vitamin 1724 volatile biomarker 542 vorinostat 1391, 1680

WASF3 1066 wholegrain products 730 Wnt signalling 910 *WT1* 1255

xenograft 43, 498, 1588 Xuanwei cohort 727

YM155 36

Zoledronic acid 629

